# Determinants of Cell- and Gene-Specific Transcriptional Regulation by the Glucocorticoid Receptor

**DOI:** 10.1371/journal.pgen.0030094

**Published:** 2007-06-08

**Authors:** Alex Yick-Lun So, Christina Chaivorapol, Eric C Bolton, Hao Li, Keith R Yamamoto

**Affiliations:** 1 Department of Cellular and Molecular Pharmacology, University of California San Francisco, San Francisco, California, United States of America; 2 Chemistry and Chemical Biology Graduate Program, University of California San Francisco, San Francisco, California, United States of America; 3 Department of Biochemistry and Biophysics, University of California San Francisco, San Francisco, California, United States of America; 4 California Institute for Quantitative Biomedical Research, University of California San Francisco, San Francisco, California, United States of America; 5 Graduate Program in Biological and Medical Informatics, University of California San Francisco, San Francisco, California, United States of America; Lawrence Livermore National Laboratory, United States of America

## Abstract

The glucocorticoid receptor (GR) associates with glucocorticoid response elements (GREs) and regulates selective gene transcription in a cell-specific manner. Native GREs are typically thought to be composite elements that recruit GR as well as other regulatory factors into functional complexes. We assessed whether GR occupancy is commonly a limiting determinant of GRE function as well as the extent to which core GR binding sequences and GRE architecture are conserved at functional loci. We surveyed 100-kb regions surrounding each of 548 known or potentially glucocorticoid-responsive genes in A549 human lung cells for GR-occupied GREs. We found that GR was bound in A549 cells predominately near genes responsive to glucocorticoids in those cells and not at genes regulated by GR in other cells. The GREs were positionally conserved at each responsive gene but across the set of responsive genes were distributed equally upstream and downstream of the transcription start sites, with 63% of them >10 kb from those sites. Strikingly, although the core GR binding sequences across the set of GREs varied extensively around a consensus, the precise sequence at an individual GRE was conserved across four mammalian species. Similarly, sequences flanking the core GR binding sites also varied among GREs but were conserved at individual GREs. We conclude that GR occupancy is a primary determinant of glucocorticoid responsiveness in A549 cells and that core GR binding sequences as well as GRE architecture likely harbor gene-specific regulatory information.

## Author Summary

The glucocorticoid receptor (GR) regulates a myriad of physiological functions, such as cell differentiation and metabolism, achieved through modulating transcription in a cell- and gene-specific manner. However, the determinants that specify cell- and gene-specific GR transcriptional regulation are not well established. We describe three properties that contribute to this specificity: (1) GR occupancy at genomic glucocorticoid response elements (GREs) appears to be a primary determinant of glucocorticoid responsiveness; (2) the DNA sequences bound by GR vary widely around a consensus, but the precise sequences of individual GREs are highly conserved, suggesting a role for these sequences in gene-specific GR transcriptional regulation; and (3) native chromosomal GREs were generally found to be composite elements, comprised of multiple factor binding sites that were highly variable in composition, but as with the GR binding sequences, highly conserved at individual GREs. In addition, we discovered that most GREs were positioned far from their GR target genes and that they were equally distributed upstream and downstream of the target genes. These findings, which may be applicable to other regulatory factors, provide fundamental insights for understanding cell- and gene-specific transcriptional regulation.

## Introduction

The great challenge of metazoan transcriptional regulation is to create specialized expression pathways that accommodate and define myriad contexts, i.e., different developmental, physiological, and environmental states in distinct organs, tissues, and cell types. This is achieved by a network of transcriptional regulatory factors, which receive and integrate signaling information and transduce that information by binding close to specific target genes to modulate their expression. For example, the glucocorticoid receptor (GR) associates selectively with corticosteroid ligands produced in the adrenal gland in response to neuroendocrine cues; the GR-hormone interaction promotes GR binding to genomic glucocorticoid response elements (GREs), in turn modulating the transcription of genes that affect cell differentiation, inflammatory responses, and metabolism [1,2]. Expression profile analyses have identified glucocorticoid responsive genes in different cell types [3,4], and it is striking that there is only modest overlap in glucocorticoid-regulated gene sets between two cell types. The mechanisms by which GR selectively regulates transcription in cell-specific contexts are not well established.

An intriguing feature of GREs and other metazoan response elements is that their positions relative to their target genes are not tightly constrained [5,6]. Although certain metazoan response elements have been described that operate from long range, most searches for such regulatory sequences have nevertheless focused for technical reasons on restricted zones just upstream of promoters, where prokaryotic and fungal elements reside. Thus, the GRE for interleukin-8 *(IL8)* is just upstream of the promoter [7], whereas the tyrosine aminotransferase GRE resides at −2.5 kb [8]. Recent, more systematic searches for response elements have revealed dramatic examples, such as an estrogen response element 144 kb upstream from the promoter of the *NRIP* gene [9], and an intragenic region 65 kb downstream from the *Fkbp5* promoter that appears to serve as an androgen response element [10]. It has been suggested that long-range regulatory mechanisms are likely to facilitate and promote regulatory evolution [11]. However, it has not been determined whether the position of a response element relative to its target gene is functionally significant.

Evidence from numerous anecdotal, gene-specific studies indicates that native response elements are typically composite elements that encompass distinct sequence motifs recognized by two or more regulatory factors. In turn, the bound factors recruit non-DNA binding coregulatory factors, forming functional regulatory complexes that remodel chromatin and modify the activity of the transcription machinery. In this scheme, the structure and activity of the regulatory complex at a given response element would be specified by at least three determinants: the sequence motifs comprising the response element; the availability of those sequences for factor binding; and the availability and activity levels of regulatory factors present in the cell. For example, primary GREs, defined as those at which GR occupancy is required for glucocorticoid-responsive regulation, are a diverse family of elements that bind GR together with an array of additional factors defined by the above three determinants. Such composite response elements provide a powerful driving force for combinatorial regulation [2,12], vastly increasing the capacity of a single factor to assume multiple regulatory roles. Indeed, the mere presence of GR in a regulatory complex is not sufficient for glucocorticoid regulation [7]. It is not known, however, whether such “nonproductive” binding by GR is common, or if instead GR occupancy is a strong indicator of GRE function.

GR binds to a family of related sequences that defines a consensus motif: an imperfect palindrome of hexameric half sites separated by a three-bp spacer [13–15]. Within those 15-bp core GR binding sequences, a few positions are nearly invariant, whereas a substantial proportion can be altered with little effect on GR binding affinity [16]. However, the functional consequences of such “permitted” sequence variations are unknown. GR can mediate a range of regulatory processes within a single cell type, including activation and repression of specific genes [4,17]. These findings, together with the results of biochemical and structural studies, raise the possibility that the core GR binding sequences might themselves serve as distinct “GR ligands,” allosterically affecting GR structure to produce distinct GR functions [18,19]. Studies of other regulatory factors have led to similar conclusions [20,21]. If different core GR binding sequences indeed produce GRE-specific (and therefore target gene-specific) regulatory activities, we could expect that the core GR binding sequence associated with a given target gene would be strongly conserved through evolution, whereas the collection of core GR binding sequences across different genes would vary substantially. Analogously, if the architecture of composite GREs, i.e., the arrangements of additional sequence motifs surrounding the core GR binding site, are also important for gene-specific regulation, we would expect flanking sequences surrounding the core GR binding site to also be evolutionarily conserved in a GRE-specific manner but not across GREs within a single genome. Neither of these notions has been examined.

In the present work, we sought to define and characterize a set of GREs in A549 human alveolar epithelial cells. Thus, we determined in A549 cells the presence of GR at specific GREs close to genes that are steroid regulated across a range of cell types. We assessed whether the GR-occupied GREs were limited mainly to genes that are GR regulated in A549 and measured within and between species the conservation of GRE sequences, architecture, and genomic positions.

## Results

### Identification of GR Binding Regions Using Chromatin Immunoprecipitation-Microarray

To assess the correlation of GR occupancy with glucocorticoid responsiveness, we examined GR binding at three classes of genes in A549 human lung carcinoma cells: first, genes regulated by GR in A549 cells; second, genes regulated by GR in U2OS human osteosarcoma cells but not in A549; third, genes regulated by GR or the androgen receptor (AR) in cells other than A549 or U2OS. The AR-responsive genes were of interest because AR is closely related to GR and shares similar DNA-binding specificity in vitro [14,16,22]. The first two classes of genes were identified in our lab using expression microarrays, whereas the third class was compiled from our own microarray data and from published reports of others [3,4,23,24]. Both positively and negatively regulated genes were included; together the three classes comprised 548 candidate GR target genes. By examining these genes for GR binding in A549 cells, we could determine if GR occupancy in vivo is restricted only at genomic sites of genes actually regulated by glucocorticoids in A549 cells; alternatively, GR might also bind at genes that are not under glucocorticoid control in A549, but are regulated by GR or AR in other cells.

**Figure 1:**
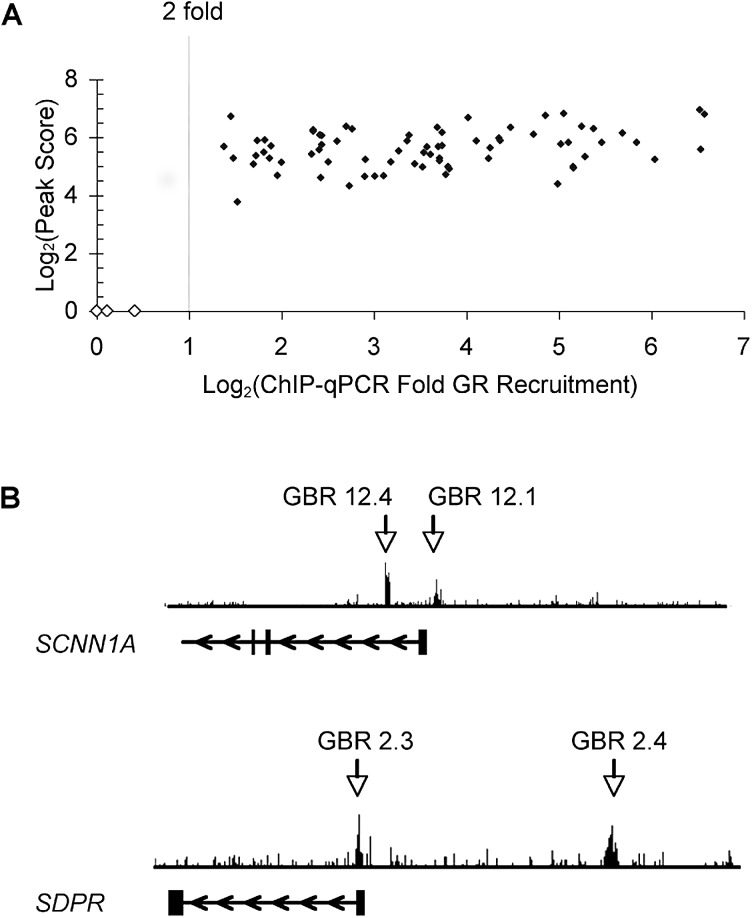
ChIP-Chip Identified Known and Novel GR Binding Regions (A) Identification of GBRs. The Log_2_(peak score) of GBRs obtained from the ChIP-chip arrays is plotted versus the dex-induced enrichment of GR at the corresponding GBR, which was assessed by GR ChIP-qPCR (averaged over at least three independent experiments. Note: dex-induced GR binding at the GBRs reproducibly in all the individual experiments). Solid diamonds, bona fide GBRs; open diamonds, negative control regions. *(*B) GBRs identified near *SCNN1A* and *SDPR* genes. Vertical bars, exons; horizontal lines, introns; arrows, direction of transcription. GBRs 12.1 and 2.3 are known promoter proximal GBRs associated with *SCNN1A* and *SDPR,* respectively. GBRs 12.4 and 2.4 are novel GBRs identified in the present work. GBR nomenclature: unique identifiers corresponding to the human chromosome number containing the GBR followed by an arbitrary integer tag.

To identify GR binding regions (GBRs), we used chromatin immunoprecipitation-microarray (ChIP-chip) to interrogate 100-kb genomic segments centered on the transcription start sites (TSSs) of our set of 548 genes. This ~55-Mb sample of the genome also included or impinged upon an additional 587 genes not previously reported to be regulated by GR; thus, we assessed GR occupancy in the vicinity of more than 1,000 genes. Immunoprecipitated chromatin samples from A549 cultures treated for one hour with the synthetic glucocorticoid dexamethasone (dex) (100 nM) or ethanol were hybridized onto the ChIP-chip tiling arrays. Independent biological replicates were hybridized onto two separate arrays, and GBRs were identified using the SignalMap detection program; we detected a 3.4% false positive rate for the GBRs found in both arrays as assessed by conventional ChIP and quantitative PCR (qPCR) analysis. Importantly, we did not detect GR occupancy at 22 regions that showed no GR binding in the arrays (unpublished data). The ChIP-chip experiments revealed a total of 73 GBRs adjacent to 61 genes (Table 1), which were validated by GR ChIP and qPCR analysis (Figure 1A). In addition to identifying GBRs previously detected by conventional ChIP, our experiments revealed novel GBRs in regions not searched in prior studies. For example, two known promoter proximal GBRs at SCNN1A [25] and SDPR [3] were confirmed in the ChIP-chip arrays as well as newly observed GBRs +3 kb and −20 kb from the SCNN1A and SDPR TSSs, respectively (Figure 1B).

**Table 1:**
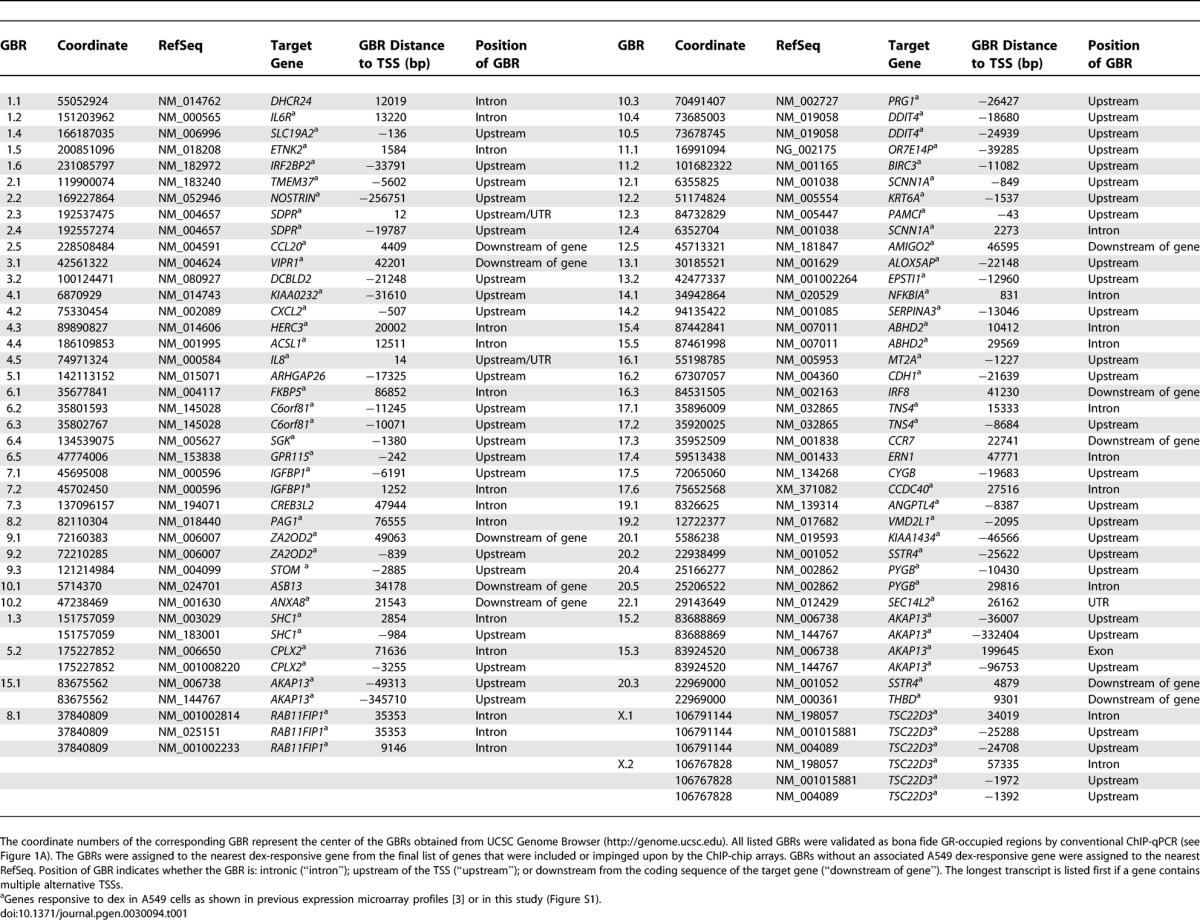
Relative Locations of GBRs

### GR Occupancy Correlates with Glucocorticoid Responsiveness

**Figure 2:**
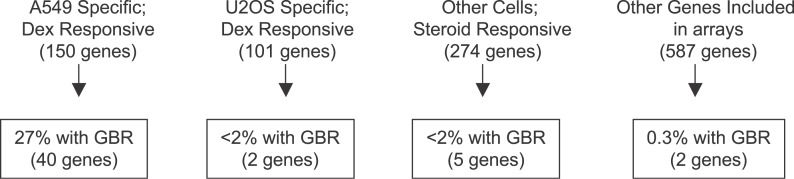
Percentage of Genes Associated with One or More GBRs in A549 Cells A549-specific dex-responsive genes are regulated by GR in A549 cells but not U2OS cells. U2OS-specific dex-responsive genes are regulated by GR in U2OS cells but not in A549 cells. The 34 genes regulated by GR in both A549 and U2OS cells, 12 of which associated with an A549 GBR, were excluded from the analysis shown. Genes responsive to glucocorticoids or androgens in cells other than A549 and U2OS are denoted as “other cells steroid responsive.” Lastly, additional genes that were wholly or partially included in our ChIP-chip arrays due to the extensive sampling of regions around all the genes mentioned above are represented as “genes included in arrays.”

Of the 73 A549 GBRs identified in the present study, 64 (88%) were associated with genes regulated by GR in those cells (Table 1). Although the remaining nine GBRs may be nonfunctional, they may mediate responses under different biological conditions. Notably, 27% of the genes that were glucocorticoid responsive specifically in A549 but not in U2OS cells were associated with a GBR, whereas only 1.9% of the genes responsive to glucocorticoids in U2OS but not in A549 contained A549 GBRs (Figure 2). Similarly, only 1.8% of the genes that were glucocorticoid or androgen responsive in other cells and only 0.3% of the genes that were sampled by the ChIP-chip arrays but were not steroid regulatory targets were associated with A549 GBRs (Figure 2; Table S1). Thus, GR occupancy in A549 cells is generally restricted to genes that are actually regulated by glucocorticoids in those cells; specifically, GR is rarely bound in A549 cells at genes responsive to glucocorticoids in other cells. We conclude that GR occupancy is a major determinant of glucocorticoid responsiveness in A549 cells at the genes assessed in this study.

### A549 GBRs Are Functional GREs

**Figure 3:**
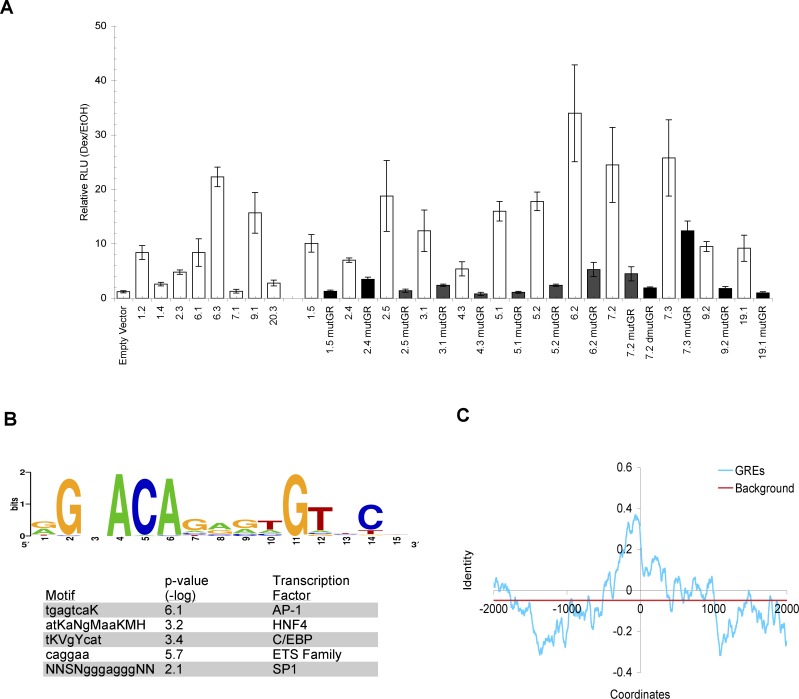
Native Chromosomal GREs Are Composite Elements (A) GBRs confer glucocorticoid responsiveness. A549 cells transfected with luciferase reporter genes linked to 500 bp GBRs were treated with EtOH or 100 nM dex for 5–7 h, harvested, and measured for luciferase activity. Fold dex inductions are plotted for wildtype (white) reporters and mutant (black) reporters with singly (mutGR) or doubly mutated (dmutGR) GR binding sites; standard errors of mean over at least three independent experiments are shown. The 13 mutated GR binding sites were randomly chosen. The GREs that harbor these GR binding sites represent a range of enriched GBRs, ranging from ~6- to 40-fold dex-induced GR occupancy as assessed by ChIP-qPCR (unpublished data). (B) Identification of enriched motifs within GBRs is shown. Top panel: Sequence logo, generated using WebLogo [58], represents all the compiled sequences resembling GR binding sites identified through computational analysis. Bottom panel shows other enriched motifs (displayed in IUPAC symbols) found in the GRE sequences. Motifs resembling AP-1, HNF4, and C/EBP binding sites were identified using BioProspector whereas motifs similar to ETS and SP1 binding sites were found with MobyDick. The *p*-values of the enriched motifs represent the random probability of these motifs occurring within the GREs. (C) Conservation analysis of GREs. The identity of the human and mouse sequences was calculated as number of bp matches minus the number of bp deletions or insertions, divided by a 50-bp window. Shown are the average identities for each window across 50 GREs. The background level was calculated as the average of all conservation scores across the 4-kb region. The abscissa shows bp positions with 0 defined as the center of core GR binding sites for GREs.

To test whether the A549 GBRs can confer glucocorticoid-directed transcriptional responses, we cloned 500-bp DNA fragments encompassing the GBRs into luciferase reporter plasmids. Of the 20 GBRs randomly selected from the 73 GBRs identified in this study, 19 were dex responsive in A549 cells as assessed by reporter analysis (Figure 3A). We define primary GREs (denoted here simply as GREs) as genomic regions that are occupied in vivo by GR and confer glucocorticoid-regulated transcription in transfected reporters. Although the reporter analyses do not prove that the identified elements are functional in their native contexts (see Discussion), they establish that the 500-bp fragments tested harbor sufficient information for GR to regulate transcription. Thus, we shall refer to the GBRs henceforth as GREs.

### GREs Are Generally Distal and Evenly Distributed between Upstream and Downstream Regions

**Figure 4:**
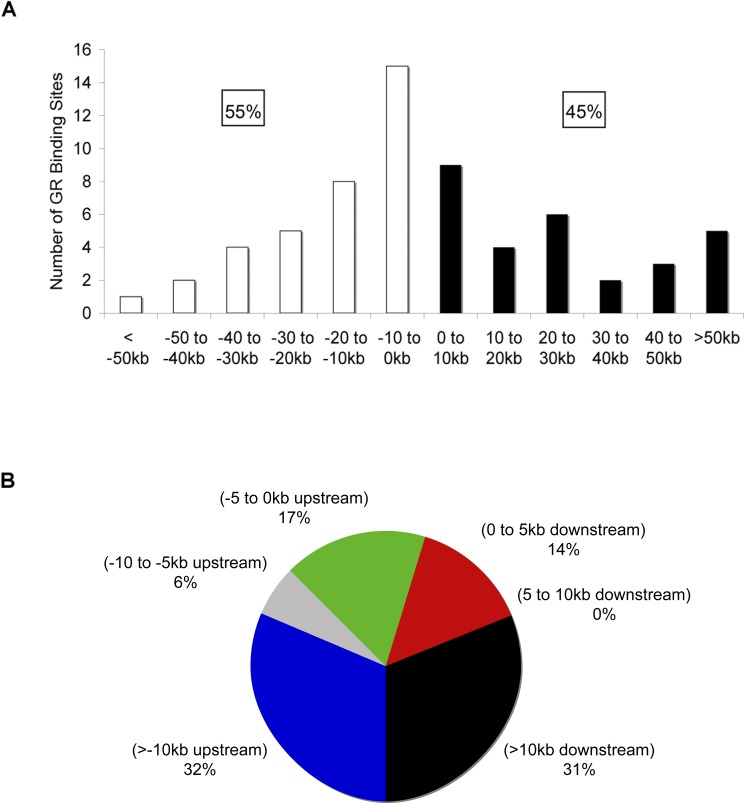
Location and Position of GREs (A) Locations of GREs relative to the TSS of target genes. The number of GREs resident in 10-kb increments relative to the TSS of the target gene are plotted. White bars and black bars represent GREs upstream and downstream of the TSS, respectively. (B) Distribution of GREs relative to target gene transcription start site is shown. The chart presents percentage of GREs at various positions upstream and downstream of target genes. Note that only 64 of the 73 GREs detected in A549 cells were included in these analyses; the remaining nine GREs did not associate with a dex-responsive gene in these cells. The GREs were assigned to the nearest gene regulated by GR in A549 cells from the final list of genes that were included or impinged upon by the ChIP-chip arrays**.** Coordinates of TSSs were obtained from UCSC Genome Browser based on RefSeq. Similar results were obtained when we used TSS coordinates that were experimentally determined (DataBase of Transcriptional Start Sites) through 5′ end cloning (unpublished data) [59]. The TSS of the longest transcript was used for genes that have multiple alternative TSSs. Similar results were obtained if the GREs were assigned the closest TSS of the associated dex-responsive gene: 38% of GREs were located downstream from TSS; 58% of GREs were positioned farther than 10 kb from the assigned TSSs.

We determined the positions of the A549 GREs relative to TSSs of their respective target genes (Figure 4A). For this analysis, the GREs were assigned to the nearest gene responsive to dex in A549 cells. Surprisingly, we found that 45% of the GREs were located downstream of the TSSs, suggesting that GR exhibits transcriptional regulation without a significant preference for regions upstream or downstream of TSSs (Table 1). Figure 4B summarizes the distribution of promoter proximal (within 5 kb from the TSS) and distal GREs (farther than 10 kb from the TSS). Strikingly, 63% of the GREs were distal, whereas only 31% of them were promoter proximal (Figure 4B). Mammalian response elements are commonly thought to reside upstream and proximal to their cognate promoters; thus, identification of GREs and response elements in general have mainly focused on these regions. Importantly, Figure 4B demonstrates that only a small fraction of the GREs (17%) identified in this study was positioned within these regions. These results indicate that GREs are just as likely to be located downstream of the TSSs and that the majority operate remotely from their target promoters, at least by linear DNA distance.

**Figure 5:**
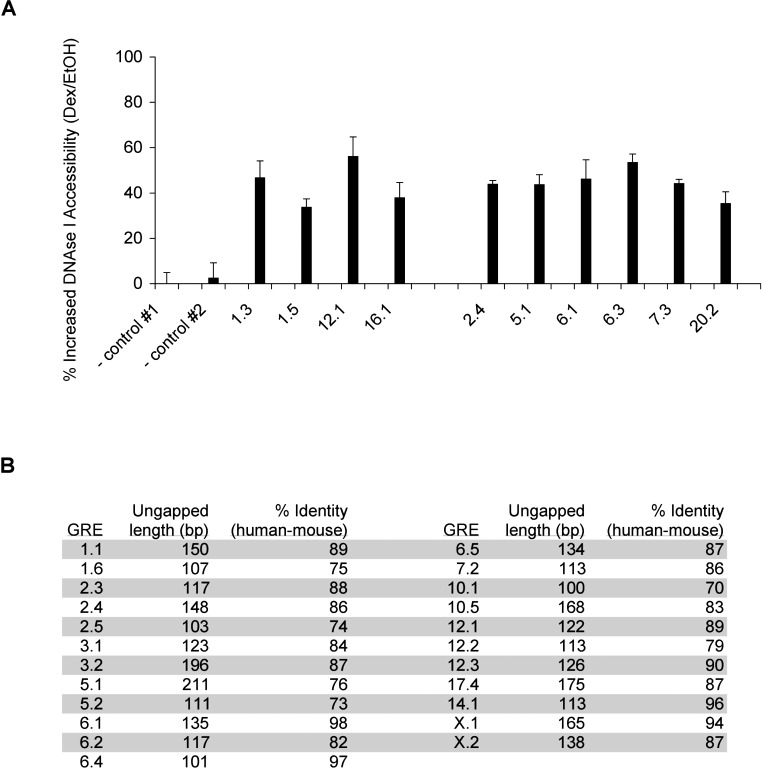
Some GREs Are Conserved Nongenic Sequences That Exhibit Increased DNAse I Accessibility Upon GR Occupancy (A) Dex induces increased DNAse I accessibility. Nuclei from A549 cells treated with EtOH or dex for 1 h were isolated, treated with DNAse I, and harvested for DNA. The relative amount of the DNA at the corresponding region were assessed by qPCR and presented as percent cleavage, with standard error of mean averaged among at least three independent experiments. Controls #1 and #2 correspond to regions near *AMOTL2* and *CDH17* genes, respectively, which do not exhibit dex-induced GR occupancy (unpublished data). (B) Human–mouse sequence conservation within GREs is presented. The mouse sequences aligned with 500-bp human GRE sequences were obtained from UCSC Genome Browser. The lengths of the continuous GRE sequences without gaps between human and mouse are shown, and the percent identity of these regions were calculated as number of matched base pairs divided by length of fragment.

**Figure 6:**
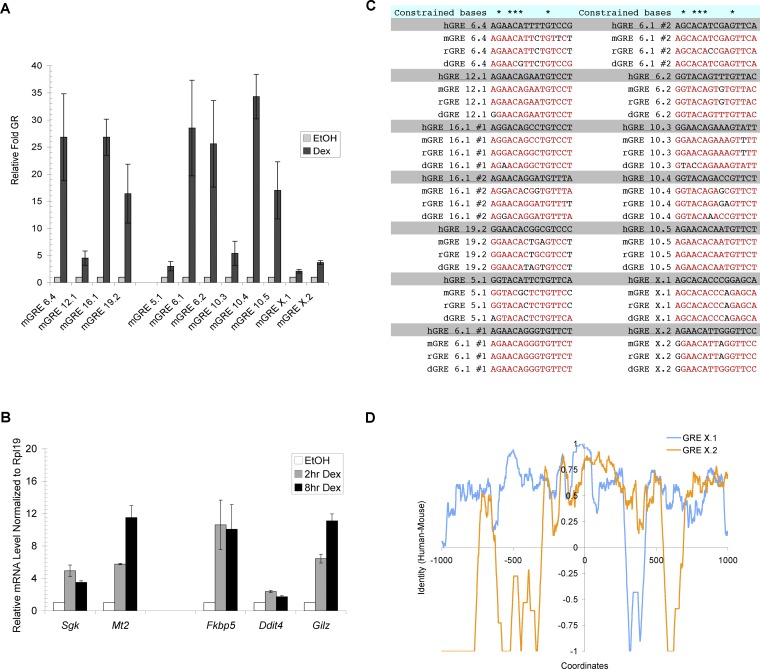
Sequence of GREs as Determinants of Gene-Specific Transcriptional Regulation by GR (A) Binding of GR at mouse orthologs of primary GR target genes from human A549 cells is shown. ChIP experiments were performed to monitor GR binding in EtOH and dex-treated C3H10T1/2 cells at genes shown. Immunoprecipitated DNA samples were analyzed with qPCR and normalized to a region near the mouse *Hsp70* gene. The nomenclature mGRE represents GRE sequences detected in the mouse genome. (B) Genes adjacent to GREs are regulated by GR in C3H10T1/2 cells. Reverse transcribed RNA samples (cDNA) from C3H10T1/2 cells treated with EtOH or 100 nM dex were subjected to qPCR and normalized to mouse *Rpl19* transcripts. (C) Core GR binding sequences are highly conserved. GR binding sequences from human (h), mouse (m), rat (r), and dog (d) are shown. Red sequences represent bases that are identical to that of human. Note that GREs 6.1 and 16.1 each contain two GR binding sites. *(*D) Comparative sequence conservation across individual GREs. Sequence identities between human and mouse of GRE X.1 and X.2 were obtained using the same calculation as Figure 3C. The coordinates represent bp positions with 0 defined as the center of core GR binding sites.

Our finding concerning GRE distribution is supported by two indirect analyses using nuclease sensitivity and sequence conservation. Sabo et al. found that DNAse I hypersensitive sites, indicative of chromatin-bound factors, are broadly distributed with a majority located >10 kb from the nearest TSS [26]. Furthermore, Dermitzakis et al. showed that conserved nongenic sequences (CNGs), ungapped 100-bp fragments with at least 70% identity between human and mouse that are presumed factor-binding regions, have no significant preference for promoter proximal regions [27,28]. As expected [29], we found that GR occupancy was correlated with DNAse I-hypersensitive cleavage at both promoter proximal (1.3, 1.5, 12.1, and 16.1) and distal GREs (2.4, 5.1, 6.1, 6.3, 7.3, and 20.2) (Figure 5A). In addition, by aligning the human GRE sequences with the corresponding regions in the mouse genome, we found that 23 of the GREs correspond to CNGs (Figure 5B). Moreover, GR occupancy and glucocorticoid responsiveness for several of these GREs/CNGs (6.4, 12.1, 5.1, 6.1, 6.2, 10.5, X.1, and X.2) were maintained in mouse cells (see Figure 6A, 6B). Thus, by testing the GREs identified in our study, we were able to provide direct support for the notion that DNAse I hypersensitive sites and CNGs serve as regulatory elements [26,28].

### Computation and Conservation Suggest That Native GREs Are Composite Elements

Native GREs, defined as naturally evolved genomic elements that confer glucocorticoid regulation on genes in their chromosomal contexts, are likely to be “composite elements,” made up of binding sites for GR together with multiple nonreceptor regulatory factors [2]. To assess whether we could detect such complex architecture, we used computational approaches (Bioprospector and MobyDick) to survey the 500-bp GRE-containing fragments for sequences related to known regulatory factor binding sites [30–32]. The most prominent motif found, present in 68% of the GRE sequences, was a series of imperfect palindromes similar to known core GR binding sites (Figure 3B). Potentially, GR may interact with the remaining 32% of GREs through other recognition motifs or through tethering to other factors [7]. Mutagenesis of computationally predicted core GR binding sites decreased or completely abolished dex stimulation for each of 13 randomly tested sites, validating this approach for identifying functional core GR binding sequences (Figure 3A). Some GREs, such as 6.2, 7.2, and 7.3, contained multiple GR binding sites; we found that reporters mutated at only one of those sites retained residual dex inducible activity. These experiments imply that most of the core GR binding sites identified in our computational analysis are functional.

In addition, we found that motifs similar to AP-1, ETS, SP1, C/EBP, and HNF4 binding sequences were enriched in the 500-bp GRE fragments (Figure 3B). For example, motifs resembling AP-1 and C/EBP binding sites were identified in the GRE of the *IL8* gene. Importantly, the AP-1 binding site is known to be crucial for regulation of *IL8* by the AP-1 factor [33]; similarly, C/EBPα enhances transcription of a reporter spanning the *IL8* GRE region [34]. Thus, as with GR binding sequences, our computational analysis was capable of discovering functional nonreceptor binding sites. Detection of multiple factor binding sites within the GRE sequences is consistent with the hypothesis that native GREs are typically composite response elements that recruit heterotypic complexes for combinatorial control [2].

To estimate the extent of GRE conservation, we measured sequence identity in human and mouse across 4-kb regions centered on the core GR binding sites (see Figure 3C legend and Materials and Methods) averaged across 50-bp windows; a similar (albeit higher resolution) pattern was obtained with 15-bp windows (unpublished data). Strikingly, we found that flanking sequences roughly 1 kb surrounding the core GR binding sites were conserved relative to background (Figure 3C). This elevated evolutionary conservation implies that these segments are biologically functional, not only in reporter constructs (Figure 3A), but also in their native chromosomal contexts, further supporting the view that native GREs are composite elements.

### Sequence Conservation of Core GR Binding Sites and GREs

We next sought to examine in detail the extent of sequence conservation of some of the individual core GR binding sequences and GREs that we had identified in our study. We chose a subset of 12 human GREs that are occupied by GR both in another species, mouse, and in another cell type, C3H10T1/2 mesenchymal cells (Figure 6A). Consistent with the correlation between GR occupancy and glucocorticoid responsiveness (Figure 2; Table 1), we confirmed that several of these genes *(Fkbp5, Ddit4, Gilz, MT2A,* and *Sgk)* were indeed dex inducible in the C3H10T1/2 cells (Figure 6B). These 12 GREs resided at very different locations relative to the TSSs of their human target genes (ranging from 0.1 kb to 86 kb) (Table S2); remarkably, however, each locus was approximately maintained in the mouse genome (Table S2). This finding suggests that the positions of individual GREs may be integral to their regulatory functions.

We then examined the extent of conservation of the 15-bp core GR binding sites within the GRE set defined above. As anticipated, the 12 core GR binding sites from the different human GREs differed substantially, with only five invariant positions across the 15-bp sequences (Figure 6C); for example, the binding sites of human GRE 5.1 and human GRE 10.3 match at only seven positions. In striking contrast, we found that the core GR binding site sequences within the individual GREs were highly conserved among human, mouse, dog, and rat (Figure 6C); for example, the core GR binding sequence at GRE 10.5 is identical in all four evolutionarily distant species.

Finally, we compared in human and mouse the patterns of conserved sequences flanking the core GR binding sites, which provide “architectural signatures” of individual GREs. We found that the patterns of sequence conservation differed dramatically among the different GREs (Figure 6D; Figure S3). For example, GRE X.1 contains conserved sequence elements at −900, −500, and +600bp, whereas GRE X.2 displays no conservation at those positions (Figure 6D). Although the functional significance of the conserved regions has yet to be tested (for example, we have not ruled out incidental overlaps with conserved noncoding expressed regions), the conserved regions are likely to correspond to regulatory or structural motifs. As predicted by these findings, pair-wise calculations of sequence identity of different human GREs (using a 15-bp window centered on the core GR binding sites) demonstrated that sequences flanking the core GR binding sites varied extensively among human GREs (Figure S4). Thus, the overall family of GREs is broadly divergent in sequence and organization, but each individual GRE retains a distinctive signature of conserved sequences, suggesting that each corresponds to a composite GRE that is functionally distinct.

## Discussion

We set out to examine the organization and function of genomic elements responsible for transcriptional regulation by GR. Our study yielded five conclusions: (1) GR occupancy at a GRE is generally a limiting determinant of glucocorticoid response in A549 cells; (2) the core GR binding sequences conform to a consensus that displays substantial GRE-to-GRE variation as anticipated, but the precise binding sequences at individual GREs are highly conserved through evolution; (3) GREs appear to be evenly distributed upstream and downstream of their target genes; (4) most GREs are positioned at locations remote from the TSSs of their target TSSs; and (5) native GREs are commonly composite elements, comprised of multiple factor binding sites, and they are individually conserved in position and architecture yet very different from each other. We shall consider the implications of these conclusions in turn.

We began by surveying more than 1,000 genes, with half of them candidates for steroid regulation, and a specific subset known to be GR-regulated in A549 cells. We found that GR occupancy of A549 GREs correlated strongly (nearly 90%) with genes that are glucocorticoid responsive in A549, suggesting that GR binding is generally a limiting determinant for response in these cells. In a small number of cases, we observed GR occupancy close to genes that were GR-unresponsive in A549 cells, but were steroid regulated in other cells [4] (E. C. Bolton and K. R. Yamamoto, unpublished results). This implies that GR occupancy at these genes likely reflects bona fide response element binding, but that GR binding is not a limiting factor for glucocorticoid regulation of this minority class of genes in A549 cells. Collectively, our data suggest that restriction of GR occupancy in A549 cells may be responsible for much of the cell-specific GR-mediated regulation in these cells. The mechanisms of occupancy restriction could be positive or negative mechanisms, such as accessory factors that stabilize GR binding, or chromatin packaging that precludes it. Although the strong correlation between GR occupancy and glucocorticoid responsiveness in A549 cells seems likely to hold in other cell types, it is conceivable that responsiveness may be determined differently in other cell types. Thus, it will be interesting to examine cell-specific GR regulation in other cells to complement the observations made in A549 cells. It is intriguing that one component, GR, within such varied and complex machineries would so strongly predominate as a determinant of transcriptional regulation in A549 cells. It will be interesting to examine regulatory complexes that mediate other types of responses (e.g., heat shock and DNA damage) to assess whether response element occupancy by a single factor in each class is a dominant determinant of responsiveness.

We examined sequence conservation of a set of GREs that are occupied by GR both in human lung epithelial cells and in mouse mesenchymal stem cells. We found that the 15-bp core GR binding sequences varied greatly among the different GREs (Figure 3B), whereas the sequences of the individual binding sites were nearly fully conserved across four mammalian species (Figure 6C). Crystallographic studies demonstrate that GR makes specific contacts with only four bases of the 15-bp core binding sequence [35], yet every position, including the “spacer” between the hexameric half sites, appears to be equivalently conserved. This indicates that the binding sequences serve functions in addition to merely localizing GR to specific genomic loci and instead may carry a regulatory code that affects GR function. Leung et al. reported similarly strong evolutionary conservation of individual κB binding sequences [36]. Indeed, Luecke and Yamamoto showed that GR directs distinct regulatory effects when tethered to NFκB at two κB response elements that differ by only one base pair [7]. Thus, one interpretation of our data findings is that factor binding sites may serve as allosteric effectors [19] in which individual binding sequences convey subtle conformational differences to specify distinct factor functions. Conceivably, this hypothesis might also explain why GR predominates as a limiting determinant of responsiveness, because factors that read allosteric regulatory codes might specify the rules for assembly of GRE-specific and thus gene-specific regulatory complexes.

To characterize the architecture of GREs, we took several approaches. In unbiased computational analyses, we identified enriched sequence motifs within 500-bp segments encompassing core GR binding sites. Sequence motifs resembling binding sites for GR, AP-1, ETS, SP1, C/EBP, and HNF4 were overrepresented relative to a background of unbound GR regions, consistent with the notion that native GREs are composite elements. For most of these GREs, the role of these factors in GR transcriptional regulation remains to be tested, but it is notable that ETS-1, SP1, and HNF4 have been shown at other genes to augment glucocorticoid responses [37–39]. Moreover, Phuc Le et al. [40] described motifs resembling AP1 and C/EBP binding sites within certain mouse GREs and showed that nearly half of the GREs predicted to encompass C/EBP binding sites did indeed bind C/EBPβ [40]. These findings further the view that our computational analysis can infer factors that potentially interact with GR at GREs. Using a similar approach, Carroll et al. [9] and Laganiere et al. [41] have interrogated estrogen response elements and identified FOXA1 as a factor playing an important role for both estrogen receptor binding and transcriptional activity. Thus, we anticipate that the factors that occupy the GR composite elements may interact physically, functionally, or both, thereby affecting binding as well as regulatory activity. Indeed, an averaged comparison of human and mouse sequences flanking core GR binding sites revealed that a region of approximately 1 kb was conserved above the background level (Figure 3C), suggesting that native composite GREs are extensive and typically may contain numerous factor binding sites. Interestingly, individual GREs displayed distinctive patterns of sequence conservation extending from the core GR binding sites (Figure 6D; Figure S3). These GRE signatures likely reflect conservation of various sequence motifs at different positions within each element, producing GRE-specific (and therefore gene-specific) architecture that likely creates distinct regulatory effects.

To investigate the distribution of regulatory elements relative to their target genes, we monitored GR occupancy across 100 kb regions centered on the TSSs of glucocorticoid responsive genes. We found that GREs were evenly distributed upstream and downstream of their target genes with the majority located >10 kb from their target promoters; other metazoan regulatory factors, such as estrogen receptor (ER) and STAT1, have similarly been reported to act from sites remote from their target genes [9,42–45]. In contrast to these factors, E2F1 was shown to mainly bind promoter proximal regions [42]; others have used computational approaches to infer factor binding sites close to promoters, but these have not been experimentally confirmed [46]. In parallel with our findings, Carroll et al. reported that only 4% of estrogen receptor ER binding regions was mapped within −800 bp to +200 bp from TSS of known genes from RefSeq [43]. Our data demonstrated that 9% of GBRs were positioned at this location. These studies together imply that steroid receptors, which include estrogen receptor and GR, in general regulate transcription from remote locations. Interestingly, we found that the positions of individual GREs were generally conserved across species (Table S2), implying that GRE position may be functionally important for target gene regulation. In any case, our findings differ dramatically from those in prokaryotes and fungi, where transcriptional regulatory elements are promoter proximal. It has been suggested that these two broad classes of regulatory mechanisms, so-called long range and short range, are mechanistically and evolutionarily related, and that long range control might facilitate regulatory evolution [11]. As predicted by that model, distal elements, far from target genes as measured by linear DNA distance, may operate in close proximity with their target promoters in 3-D space*.* For example, Carroll et al. detected an interaction between the *NRIP-1* promoter and its distal estrogen response element [9]. It will be interesting to determine whether response element location (i.e., promoter proximal versus distal) is somehow related to mechanism or to physiological network.

Remote response element locations can complicate assignment of cognate target genes. An extreme example is olfactory receptor gene expression, which is governed by a regulatory element that can operate on target genes located on different chromosomes [47]. In this study, we assigned the GREs to the nearest RefSeq gene responsive to dex in A549 cells. In other contexts, these GREs may be nonfunctional or may operate on genes other than those assigned in A549 cells (Table 1). Clearly, unequivocal assignment of a GRE to a given target gene will require genetic manipulations not readily accessible in mammalian cells at present. It is encouraging, however, that GR occupancy of GREs correlated strongly with glucocorticoid responsiveness of adjacent genes, supporting the view that these are bona fide direct GR targets (Figure 2; Table 1). In fact, when these genes were subjected to Gene Ontology analysis, we found that they were enriched in cell growth and immune responses (unpublished data), two biological processes regulated by GR in A549 cells [48,49]. We found GR occupancy at genes up- and down-regulated in response to dex, consistent with GR serving either as activator or repressor in different contexts. At present, we cannot assess the significance of the finding that GR was detected at GREs adjacent to activated genes versus repressed genes at a 6:1 ratio in A549 cells; whether this difference reflects differences in GRE occupancy, epitope accessibility, crosslinking efficiency, or other variables has not been determined.

Genomic response elements orchestrate transcriptional networks to mediate cellular processes for single- and multicellular organisms. The present study advanced our understanding of the organization, evolution, and function of GREs and at the same time raised a series of interesting questions. Among the more intriguing: How is GR occupancy restricted to a small subset of potential GREs in a given cell context? What is driving the strong conservation of virtually every base pair within the core GR binding sequence at individual GREs? Addressing these and other questions raised in our study will contribute additional new insights about gene regulation by GR and by other regulatory factors.

## Materials and Methods

### Cell culture, plasmids, and reporter analysis.

A549 and C3H10T1/2 cells were grown in DMEM supplemented with 5% or 10% FBS, respectively, in a 5% carbon dioxide atmosphere. Before hormone treatment, media was replenished with DMEM containing charcoal stripped FBS, which depletes endogenous steroids. Plasmid PGL4.10 E4TATA (generously provided by Yuriy Shostak) was created by insertion of the E4TATA minimal promoter into pGL4.10 vector (Promega, http://www.promega.com). The 20 reporters tested (Figure 3A) represent randomly chosen GRE fragments. The QuikChange kit (Stratagene, http://www.stratagene.com) was used for reporter mutagenesis. The 13 core GR binding sites that were mutated in the reporters (Figure 3A) were also randomly chosen based on success of mutagenesis. GBR-containing DNA fragments (500 bp) were amplified by PCR and subcloned into pGL4.10 E4TATA using KpnI and XhoI sites (see Table S3 for primer sequences). A549 cells were grown in a 48-well plate and cotransfected with 19 ng of the reporter constructs, 10 ng pRL Luc (Promega), and 38 ng pCDNA3 hGR (human GR expression vector) using Lipofectamine 2000 (Invitrogen, http://www.invitrogen.com). After overnight transfection, cells were treated with hormone, harvested, and luciferase activity was measured as described for the dual luciferase reporter system (Promega) using a Tecan Ultra Evolution plate reader (Tecan, http://www.tecan.com).

### ChIP and array analysis.

ChIP assays were performed as described [7] with the following modifications. The chromatin samples were extracted once with phenol-chloroform and purified using a Qiaquick column as recommended by the manufacturer (Qiagen, http://www1.qiagen.com). The ligation-mediated PCR (LMPCR) process was adapted from Oberley et al. [50]. We used 3.5–20 ng of amplicon for real-time qPCR analysis, and data were normalized to *Hsp70* (see Table S4 for primer sequence). Human and mouse DNA sequences were retrieved from University of California Santa Cruz (UCSC) Genome Browser (http://genome.ucsc.edu) NCBI Build 35, and qPCR primers were designed using Primer3 [51]. For the array, ~50 kb upstream and downstream regions were tiled with isothermal 50 mer oligos (spaced on average of every 54 bp apart) relative to the TSSs of the investigated target genes. Where 100-kb regions overlapped, the surrounding genomic region was tiled further bidirectionally. ChIP samples from (final concentration, 0.01% ethanol) or dex-treated A549 cells were labeled with Cy3 or Cy5, hybridized onto the arrays, and relative signal intensities were measured by NimbleGen (http://www.nimblegen.com). SignalMap was utilized to find peak enrichments with both window threshold detection (500-bp peak window size, 25% of Peak Threshold) and second derivative peak detection (500-bp peak window size, 20 bp smooth step, 25% peak threshold) (NimbleGen).

### RNA isolation, reverse transcription, and real-time qPCR.

The RNA isolation, reverse transcription, and qPCR steps were performed as previously described [4]. Primers for cDNA amplification are displayed in Table S4.

### DNAse I accessibility assay.

The experiments were adapted from previous described protocol [52] with the following modifications. Briefly, nuclei from A549 cells treated with vehicle or dex for 1 h were treated with 6.25–200 units/ml of DNAse I (Qiagen) for 5 min at room temperature. The reaction was stopped and treated with Proteinase K for 1 h at 65 °C. The DNA samples were extracted once with 1:1 phenol-chloroform and further purified using MiniPrep columns (Qiagen). The samples were subjected to qPCR analysis to determine the relative amount of cleaved product (see Table S4 for primer sequences), which was converted to percent DNAse I cleavage.

### Computational analysis.

For computational analysis of enriched motifs, all repeat-masked DNA sequences were downloaded from the UCSC genome browser (NCBI Human Build 35). BioProspector analysis was initially performed using nucleotide widths (w) 14 and 16 on GREs to identify GR binding sites and the top motifs were masked to identify other motifs [32]. For MobyDick analysis, both the human and the human/mouse aligned sequences were used as inputs to identify enriched motifs [30,31]. Similar motifs were clustered using CAST [53–55]. All *p*-values for enrichment were Bonferroni corrected to identify putative factor binding sites [55]. The top Bioprospector w14 position weight matrix (PWM) was used to score GREs for putative GR binding sites with a false positive rate of less than 10%. This upper bound was calculated from randomly sampling unbound GR regions (Figure S2).

We built position weight matrices (PWMs) of those motifs with *p*-values less than 0.05, which were used to measure similarity to known binding sites in TRANSFAC [56]. We measured the distance between the PWMs and those representing binding sites for known regulatory factors using relative entropy (Kullback-Liebler divergence) with a cutoff of less than 6.0 to associate motifs with putative regulatory factor binding sites. The known binding site matrices were obtained from TRANSFAC professional version 9.3.

The human–mouse conservation score was calculated as described [9] using a 50-mer window for 50 sequences containing a putative GR binding site based on our computational and experimental analysis (Figure S2 and Figure 3A). The conservation score was calculated as number of bp matches minus the number of bp deletions or insertions divided by the bp window size. We centered each alignment based on the highest scoring putative GR binding site in human and expanded equally on each side of the binding site to a total length of 4 kb. The background level was calculated by taking the average of all conservation scores across the 4-kb region. The human(hg)/mouse(mm) genome alignments were downloaded from Vista (http://pipeline.lbl.gov/cgi-bin/gateway2).

## Supporting Information

Figure S1 Identification of Novel GR Targets in A549 Cells by ChIP-chip ChIP-chip analysis revealed GR occupancy at genes not previously recognized as GR targets in A549 cells. Analysis using qPCR demonstrated that 28 of these genes were dex responsive after four or eight hours of treatment. Induction or repression is represented as fold changes in positive or negative values, respectively, averaged over at least three independent experiments. The data shown represent largest fold change obtained from four or eight hours of hormone treatment.
(109 KB PDF)

Figure S2 Number of GREs with Putative GR Binding Sites All 73 GRE sequences were scored for putative GR binding sites using a predicted position weight matrix representative of the GR binding site. Percent of sequences predicted to contain a GR binding site with varying score cutoffs is plotted as red squares. The false positive rate (blue triangles) was calculated by randomly sampling unbound sequences at varying score cutoffs.
(126 KB PDF)

Figure S3 Sequence Conservation “Signatures” Are Distinct for Each GRE Identity scores were determined for human–mouse aligned sequences and are plotted as in Figure 6D; for clarity, data are presented as pair-wise comparisons. In (A–E) comparisons of conservation of the specified GREs are represented.
(498 KB PDF)

Figure S4 GREs Vary in Sequences (A) A sequence comparison of human GRE 10.5 with human GRE 6.1 is shown.
(B) A sequence comparison of human GRE 6.4 and X.2 is shown. The sequences were pair-wise aligned using ClustalW [57] and similarities were calculated as in Figure 3C using a 15-bp window. Coordinate 0 represents the center of the core GR binding sites. The red line represents the background level, which was calculated by taking the average of all identity scores.
(142 KB PDF)

Table S1 Dex Responsiveness of Steroid Targets from Other Cells in A549 Cells Quantification of relative mRNA levels by qPCR of a subset of the 587 genes (denoted at ChIP-chip Spanned) included in the ChIP-chip arrays (Figure 2) showed that they were not dex responsive (less than 1.6-fold change) in A549 cells after 4 or 8 h of treatment; U2OS source genes are responsive to dex in U2OS but not in A549 cells; Other cells source genes are potentially steroid responsive in other cells but not in A549 cells. Analysis with qPCR confirms that a majority of these genes were indeed not responsive to dex in A549 cells after 4 or 8 h of treatment. Values shown are fold changes comparing dex and ethanol treatment averaged over at least two independent experiments. Bold letter genes are those that are dex responsive in A549 cells.
(23 KB XLS)

Table S2 Distances of GREs Relative to Adjacent Gene Are Conserved in the Mouse Genome The distances were calculated based on coordinates of the mouse aligned GREs (mGREs) and TSSs of the GR-regulated mouse homolog genes obtained from UCSC Genome Browser. The TSS of the longest transcript was used for this calculation when a gene has multiple variants. Bold letters represent the distance of the GREs relative to the adjacent gene in mouse.
(21 KB XLS)

Table S3 Primers Used for Cloning and Mutating GRE Reporters Capitalized letters represent the restriction digestion sites used for cloning the constructs into pGL4.10 E4TATA.
(25 KB XLS)

Table S4 Primers Used for qPCR Analysis FO primer and RE primer represent the forward and reverse primer, respectively, for the corresponding amplified genomic regions or cDNA sequences of the indicated genes.
(39 KB XLS)

## Abbreviations

AR: androgen receptor
ChIP: chromatin immunoprecipitation
chip: microarray chip
CNG: conserved nongenic sequence
dex: dexamethasone
GBR: GR-binding region
GR: glucocorticoid receptor
GRE: glucocorticoid response element
*IL8,*: interleukin-8
qPCR: quantitative PCR
TSS: transcription start site
