# Supplementary material for: Determinants of Cell- and Gene-Specific Transcriptional Regulation by the Glucocorticoid Receptor
Source: PLoS Genet. 2007 Jun 8;3(6):e94. doi: 10.1371/journal.pgen.0030094 (PMC1904358; doi:10.1371/journal.pgen.0030094)
Supplement: Supplementary file 3 [file pgen.0030094.sg003.pdf]

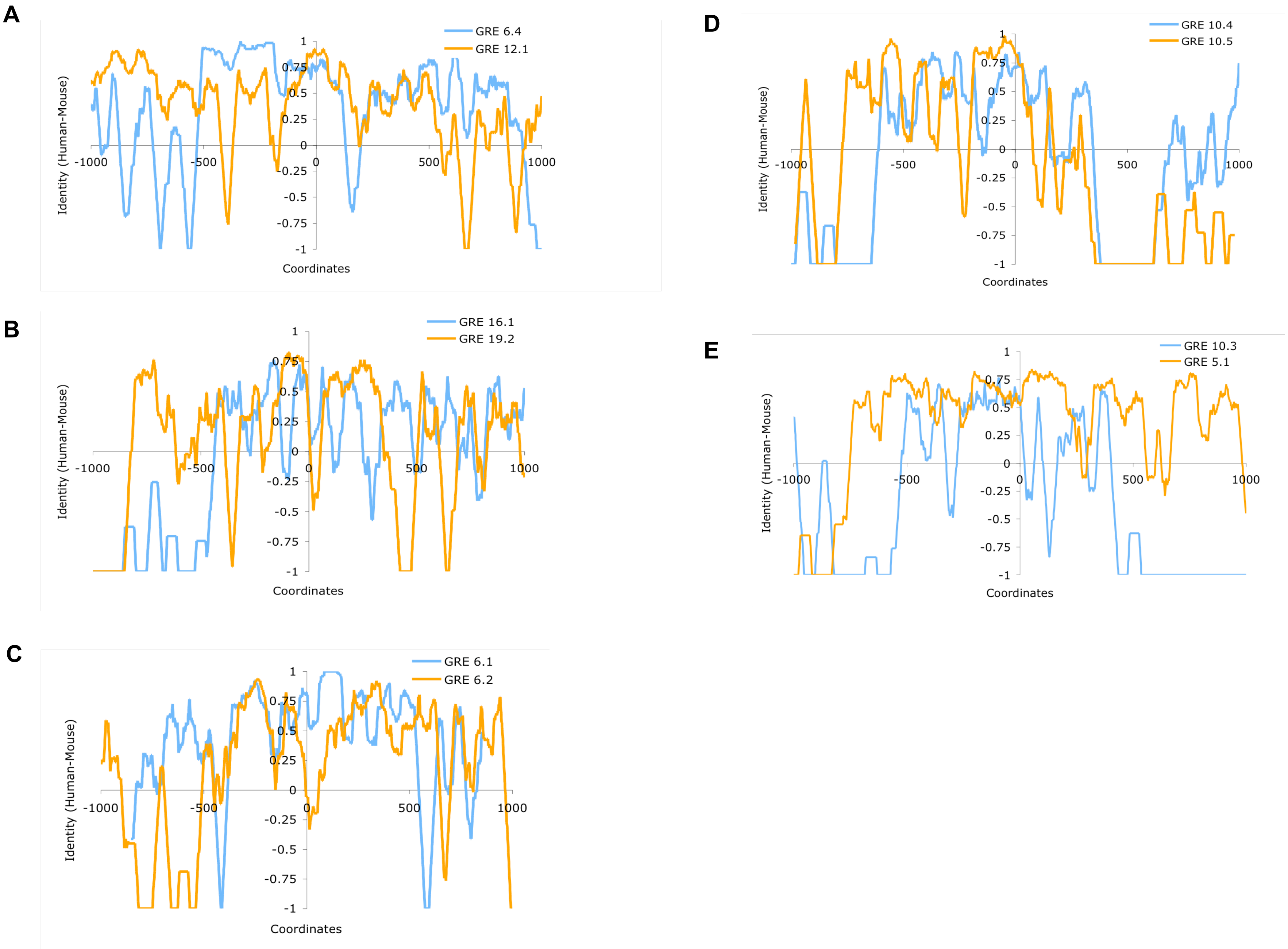

Supplemental Figure 3
